# Ceftazidime-Cefazolin Empiric Therapy for Pediatric Gradenigo Syndrome

**DOI:** 10.1177/00034894241301289

**Published:** 2024-11-20

**Authors:** Brendan K. Tao, Fahad Alotaibi, Alastair McAlpine

**Affiliations:** 1 Faculty of Medicine, University of British Columbia, Vancouver, BC, Canada; 2 Department of Paediatric Infectious Disease, BC Children’s Hospital, University of British Columbia, Vancouver, BC, Canada

**Keywords:** pediatric, Gradenigo Syndrome, abducens nerve, facial nerve pain, otomastoiditis

## Abstract

**Objective:**

Gradenigo Syndrome (GS), a rare complication of petrous apicitis secondary to acute otitis media, is characterized by (an often incomplete) triad of otorrhea, abducens nerve palsy, and facial pain along the trigeminal nerve distribution. There are several causative pathogens of petrous apicitis, including *Streptococcus* and *Staphylococcus* species, while *Pseudomonas aeruginosa* is the most common. However, the case report literature often describes antibiotic management of GS with antibiotics that do not cover *Pseudomonas*, potentially predisposing to further intracranial complications or mortality. The purpose of this work was to describe a case of pediatric Gradenigo Syndrome, successfully treated with sufficiently broad-spectrum antibiotics.

**Methods:**

This is case report.

**Results:**

A previously healthy 5-year-old boy with a history of swimming presented with esotropia and acute otitis media. Initial symptoms included otorrhea, otalgia, and pruritis, which were refractory to ciprofloxacin-dexamethasone drops. He subsequently developed a right sixth nerve palsy, suggestive of Gradenigo Syndrome, and neuroimaging showed evidence of petrous apicitis, clival osteomyelitis, and internal carotid artery stenosis. The causative organism was not elucidated to laboratory error. Given this uncertainty, he was successfully treated with empiric intravenous ceftazidime and cefazolin. After 16 weeks, he recovered fully without the need for surgery.

**Conclusions:**

In the setting of delayed or absent culture results with suspicion of skull-base infection, our case supports the use of empiric antibiotic therapy with sufficient coverage of all common pathogens including *Streptococcus*/*Staphylococcus* and *Pseudomonas aeruginosa* species, the latter of which is often not adequately covered by antibiotic regimens described in the literature.

## Introduction

Gradenigo syndrome (GS), characterized in 1904 during the pre-antibiotic era, is now a rare yet potentially life-threatening complication of acute otitis media (AOM).^1^ While GS refers to a triad of AOM, trigeminal pattern facial pain, and abducens nerve palsy, over 50% of cases are absent of the full triad.^1^ In cases of perforated AOM, the otic infection may extend to the apical petrous temporal bone, which due to its proximity to the abducens nerve (Dorello’s canal) and trigeminal ganglion (Meckel’s cave),^1^ may cause sufficient inflammation of these neural structures to precipitate the presentation of GS.^1^ Herein, we present a case of pediatric GS managed with dual ceftazidime-cefazolin therapy despite delayed ear culture results. This case demonstrates that early antibiotics with coverage of all common pathogens is necessary to prevent further complications of intracranial infection.

## Case Report

A healthy and vaccinated 5-year-old boy with history of frequent swimming presented to the British Columbia Children’s Hospital with a right esotropia and first-episode of AOM. Nine days prior, he experienced right yellow-green-colored otorrhea, otalgia, pruritis, erythema, headache, and reduced activity. He originally presented to his general practitioner who initiated ciprofloxacin-dexamethasone ear ointment, yielding symptomatic improvement. However, 6 days later, the patient complained of horizontal binocular diplopia, worse on right gaze. Optometry and ophthalmology consultation confirmed a right sixth nerve palsy, prompting his presentation to hospital.

On examination, the patient was comfortable, alert, and had normal vital signs. He had a right tympanic membrane perforation (anterior superior quadrant) with overt otorrhea, mild mastoid tenderness, and trace hearing reduction on the right side. Left ear examination was normal. The patient denied facial pain. His visual acuity was 20/20 bilaterally. Ophthalmological examination was significant for nearly full and nonpainful extraocular movements bilaterally, albeit with a mild lateral gaze deficit in the right eye without nystagmus. The rest of the ophthalmological and neurological exam was unremarkable, and there were no signs of meningitis. A review of systems was non-contributory.

Laboratory investigations exhibited a normal white blood count, marked platelet elevation (691 × 10^9^/L), and mildly raised C-reactive protein (12 mg/L). A rhinovirus/enterovirus swab was positive. Blood cultures were negative after 48 hours. However, the ear culture was found to be unprocessed after 3 days as it was lost in transit to the laboratory Repeat ear swabs and culture were negative after a further 48 hours.

**Figure 1.**
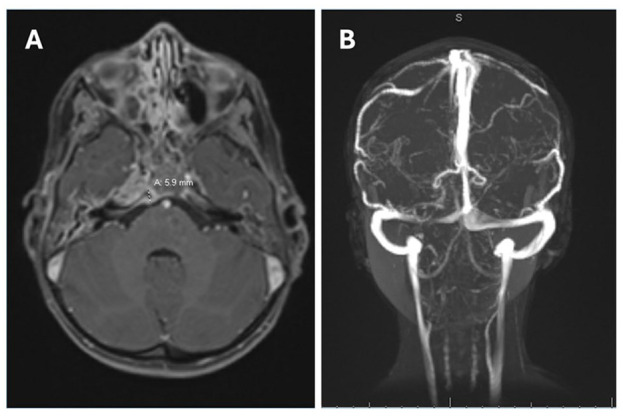
MRV head on admission. Paranasal sinus disease without invasive changes. There are bilateral middle ear and mastoid effusions with extension to the petrous apex with septal enhancement. At the rigth petrous apex and clivus, there is enhancement, patchy edema, loss of normal fine septations, and smooth osseous margins. A 5.9 mm extra-axial collection was appreciated on the right side of the prepontine cistern overlying the petroclival synchondrosis, which with associated diffusion restriction, was in keeping with a small abscess. There was asymmetric increased T2 FLAIR and postcontrast enhancement involving the wall of the petrosal and proximal cavernous segments of the right internal carotid arter with moderate luminal narrowing within these segments. No cerebral venonus sinus thrombosis was appreciated.

Magnetic resonance venography (MRV; Figure 1) on admission demonstrated otomastoiditis with coalescent right petrous apicitis, clival osteomyelitis and a 5.9 mm adjacent extradural abscess overlying the right petroclival synchondrosis. Paranasal sinus disease was noted without invasive changes. Finally, there was asymmetric enhancement involving the right carotid canal with moderate narrowing of the right internal carotid artery (ICA). No cerebral venous sinus thrombosis was evident.

Given the partial GS triad, history of frequent swimming, and petrous apicitis, intravenous ceftazidime and vancomycin were initiated for coverage of common causal pathogens (*Pseudomonas aeruginosa*, *Streptococcus pyogenes*, *Staphylococcus aureus*, and *Streptococcus pneumoniae*). Two days later, vancomycin was replaced with cefazolin to optimize bony penetrance for clival osteomyelitis. He also continued ciprofloxacin-dexamethasone drops for 10 days. His ICA stenosis, deemed secondary to acute-phase inflammation, prompted stroke prophylaxis with aspirin and 3 days of pulsed steroids (with a month-long oral taper). These treatments were well tolerated.

**Figure 2.**
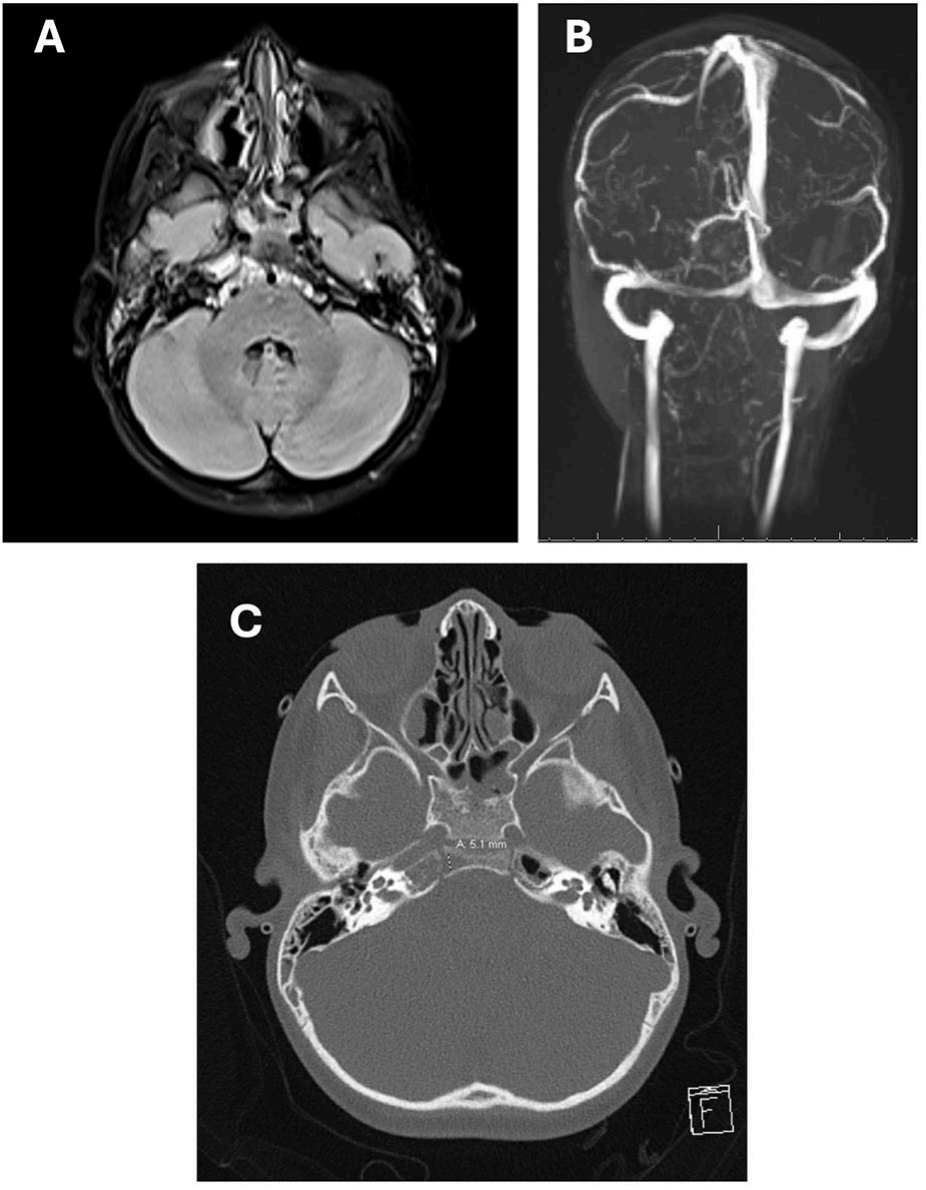
(A) MRI at follow-up week 1. There is interval improvement of the inflammatory changes involving the right petrosal-clival junction, sphenoid sinus, and right petrous apex. The small focus of diffusion restriction at the right lateral clivus, noted at admission, is no longer present. (B) There is interval improvement of the right ICA caliber likely from resolving surrounding inflammation and associated mass effect. Improved otomastoid opacification noted with some residual fluid especially at the right petrous apex. (C) CT demonstrates bony lucency within this area potentially suggestive of a small Brodie's abscess.

After 3 days, the patient’s diplopia and ocular dysmotility had resolved. Repeat neuroimaging (Figure 2) at one-week post-admission showed improved ICA caliber and osteomyelitis, but with interval development of a small Brodie’s abscess, which prompted 1 additional week of intravenous antibiotic treatment (2 weeks total). Subsequently, he completed a 4-week course of oral amoxicillin-clavulanate and ciprofloxacin. By 16 weeks post-discharge, the patient had recovered fully with no residual findings of GS, and neuroimaging showed interval resolution of the otomastoiditis, petrous apicitis, and clival osteomyelitis. Given a rapid improvement on medical therapy, surgery was deferred throughout.

## Discussion

GS is a condition with a potential for serious complications.^2^ As such, any antibiotic regimen should cover all common causative pathogens including *Pseudomonas aeruginosa* and *Streptococcus*/*Staphylococcus* species.^3^ In our case, ceftazidime and vancomycin were initiated, with vancomycin later switched to cefazolin However, in several case reports describing pediatric GS, antibiotics that cover *Pseudomonas aeruginosa*,^2,4-6^ the most common causative pathogen of petrous apicitis,^3^ are frequently omitted. Examples of such agents include (either alone or as a combination): ceftriaxone, cefotaxime, clarithromycin, vancomycin, metronidazole, benzylpenicillin, amoxicillin-clavulanate, and teicoplanin, none of which have reliable activity against *Pseudomonas aeruginosa*.^2,4-7^ Some reports utilize or suggest topical antibiotic therapies with activity against *Pseudomonas aeruginosa*,^4,6,8^ proposed to be used alongside systemic agents that cover other *Streptococcus*/*Staphylococcus* species, but this alone would not sufficiently manage cranial or intracranial infection.

Failure to adequately treat GS may result in a poor outcome. In 1 paper, the authors report a fatal outcome of GS (precluding microbiological culture) after empiric ceftriaxone use, even commenting that coverage for *Pseudomonas aeruginosa* may have been beneficial.^8^ Especially in the context of delayed culture results and clinical suspicion of skull-base involvement, a regimen with sufficient coverage of all potential common pathogens is indicated.

Intracranial complications of AOM, including GS, have seen marked incidence reductions between the pre- and post-antibiotic eras, where contemporary cases are attributable to severe initial infection, antibiotic resistance, and/or diagnostic or treatment delay. As most children will experience AOM during their lifetime, it follows that GS (while rare) can present in any specialty setting. This case underscores that clinicians should be capable of recognizing GS and expediting management, which may require multidisciplinary inpatient care. Where under-diagnosed or -treated, GS may predispose to intracranial complication, significant disability, and/or mortality. Finally, as in our case, findings of ICA involvement may warrant stroke prophylaxis.

## Conclusion

In conclusion, our case highlights the importance of early neuroimaging and microbial cultures for suspicion of GS, a rare yet potentially life-threatening complication of common AOM infections. It also emphasizes the importance of empiric systemic antibiotic therapy with activity against all common causative pathogens, including *Streptococcus*/*Staphylococcus* species and *Pseudomonas aeruginosa*, the latter of which is often inadequately covered in the case report literature.
